# *Sigesbeckia pubescens* makino alleviates ulcerative colitis in mice by modulating the Nrf2/Keap1 pathway

**DOI:** 10.3389/fphar.2025.1588525

**Published:** 2025-04-28

**Authors:** Qiu-Shuo Ma, Qi-Ling Chen, Guo-Ping Wu, Ya-Wen Yao, Yu-Xin Fan, Ke-Gang Linghu, Jun-Ming Chen, Wei Xiong, Hua Yu

**Affiliations:** ^1^ State Key Laboratory of Quality Research in Chinese Medicine, Institute of Chinese Medical Sciences, University of Macau, Taipa, China; ^2^ The High Efficacy Application of Natural Medicinal Resources Engineering Center of Guizhou Province, State Key Laboratory of Discovery and Utilization of Functional Components in Traditional Chinese Medicine & School of Pharmaceutical Sciences, Guizhou Medical University, Guiyang City, Guizhou, China; ^3^ School of Pharmacy, Shenzhen University Medical School, Shenzhen University, Shenzhen, China; ^4^ Macao Centre for Research and Development in Chinese Medicine, Institute of Chinese Medical Sciences, University of Macau, Taipa, China

**Keywords:** ulcerative colitis, siegesbeckiae herba, Sigesbeckia pubescens makino, antioxidant, Nrf2

## Abstract

**Background:**

Ulcerative colitis (UC) is a prevalent immune-mediated inflammatory bowel disease characterized by mucus secretion, hematochezia, and diarrhea. This study compared the therapeutic effects of three Siegesbeckiae Herba (SH) species used in traditional Chinese medicine—*Sigesbeckia orientalis* L (SO), *Sigesbeckia pubescens* Makino (SP), and *Sigesbeckia glabrescens* Makino (SG) — in dextran sulfate sodium (DSS)-induced UC mice.

**Methods:**

UC was induced in C57BL/6 mice with 3% DSS for 7 days. Cytokine levels in serum and colon tissues were measured by enzyme-linked immunosorbent assay. Protein and gene expression were analyzed using Western blotting and PCR. Histopathological changes were assessed via hematoxylin-eosin staining, immunohistochemistry, and immunofluorescence. Fecal specimens were collected for gut microbiota analysis. An *in vitro* UC model was also established in NCM460 cells using lipopolysaccharide (LPS), and Caco-2 cells were used to examine intestinal mucosal integrity.

**Results:**

SP substantially decreased the disease activity index, enhanced colon shortening, and mitigated histological damage in comparison to the model group. Mechanistic investigations demonstrated that SP functioned via the activation of the Nrf2/Keap1 pathway, markedly increased the activity of the antioxidant enzyme glutathione in colon tissues, decreased the concentration of the oxidative marker malondialdehyde, and upregulated the expression of the downstream genes *H O -1* and *NQO1*.

**Conclusion:**

The study reveals for the first time the differences in efficacy of different species of SH and its molecular mechanism, demonstrating that SP increases oxidative defense via the activation of the Nrf2/Keap1 pathway, therefore mitigating colitis and oxidative damage in UC mice. This discovery not only establishes a scientific foundation for the selective preference of SH species but also offers a novel technique for the creation of natural pharmaceuticals aimed at the Nrf2 pathway for the treatment of UC.

## Graphical Abstract

**Figure:**
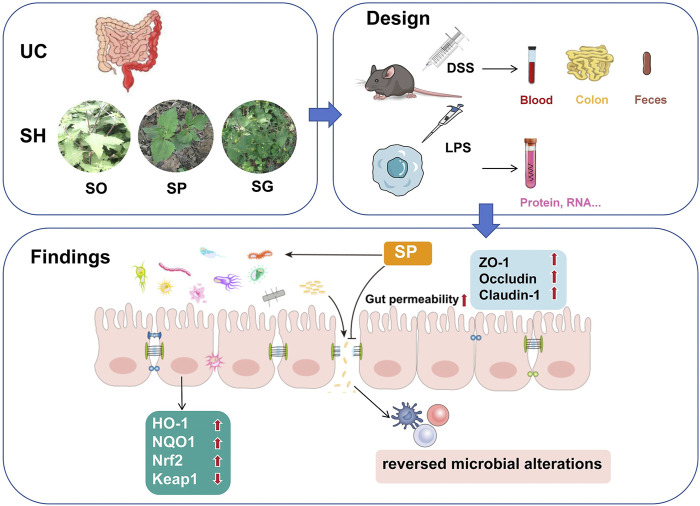

## 1 Introduction

Ulcerative colitis (UC) is a chronic inflammatory bowel disease (IBD) that primarily affects the colon and rectum, presenting with symptoms such as abdominal pain, diarrhea, and hematochezia (Ramos et al., 2023). The disease follows a relapsing-remitting course, with frequent recurrences and a prolonged disease progression. Patients with UC face an elevated risk of colon cancer, with incidence rates at least twice that of the general population (Noguchi et al., 2023). While UC was historically more prevalent in Western countries, its incidence is rising in Asia, particularly in China, where increasing urbanization, Westernized dietary patterns, and lifestyle stressors have contributed to its growing burden (Lok et al., 2008).

The precise pathogenesis of UC remains incompletely understood, but it is widely believed to result from a complex interplay of genetic predisposition, immune dysfunction, environmental factors, and alterations in the gut microbiota (Nascimento et al., 2020). Most affected patients exhibit immune dysregulation, and accompany with excessive inflammatory responses in the colon and rectum. The involvement of immunological factors is evident from clinical presentations, histopathological findings, and the efficacy of immunosuppressive therapies (Gionchetti et al., 2003; Linghu et al., 2022). UC development involves multiple immune components, including epithelial cells of the lamina propria, lymphocytes, the innate and adaptive immune systems, and the activity of various cytokines and chemokines. Moreover, results from animal studies have demonstrated that commensal gut bacteria can trigger immune alterations that contribute to UC pathogenesis (Reinecker et al., 1993, Zhao et al., 2016; Wang et al., 2025).

Amino salicylic acids, glucocorticoids, and immunosuppressants are the mainstay of UC treatment, but do not offer a complete cure and some can lead to serious adverse effects (Wu et al., 2024). The traditional Chinese medicine Siegesbeckiae Herba (SH) has long been used for managing inflammatory conditions, including arthritis and limb paralysis. Historically known as *Xixiancao* in China, it has been recommended for treating arthritis (Linghu, Zhao, et al., 2020), stroke (Chen et al., 2025), edema (Lee et al., 2016), neuro-inflammation (Chu et al., 2018; Chu et al., 2022), and gastrointestinal disorders such as diarrhea and hematochezia (Zhang et al., 2019). Modern clinical applications extend to acute enteritis (Shi, 2012). According to the *Chinese Pharmacopeia* (*1963*), the aerial part of *Sigesbeckia pubescens* Makino (SP) was originally designated as the sole source of SH, but since 1977, *Sigesbeckia orientalis* L. (SO) and *Sigesbeckia glabrescens* Makino (SG) have been included as additional sources (Ju et al., 2000). These species exhibit distinct chemical composition (Tao et al., 2018; Tao et al., 2021), resulting in varying pharmacological effects. For instance, SO has demonstrated antiallergic (Hwang et al., 2001) and immunosuppressive properties (Sun and Wang, 2006; Linghu, Xiong, et al., 2020), while kirenol, a bioactive metabolite of SH, has shown efficacy in reducing skin inflammation in murine models (Wang et al., 2011). Moreover, SP was reported to inhibit the Pam3CSK4-induced inflammation in RAW 264.7 macrophages through suppressing TLR1/TLR2-mediated NF-κB activation (Sang et al., 2018), as well as the SG could alleviate the collagen-induced arthritis in rats (Ma et al., 2020; Zhang et al., 2019). Given these variations, it is plausible that different SH species might exert differential therapeutic effects against UC.

Extensive research has focused on the development of plant-based bioactive metabolites from traditional Chinese medicine for treatment of UC (Hou and Jiang, 2013). In this study, we aimed to compare the effectiveness of SO, SP, and SG in treating dextran sulfate sodium (DSS)-induced UC in mice and to elucidate the mechanism of action of the most effective species. Our findings provide a theoretical framework for the precise selection of SH species in UC management and contribute to research on precision medicine in TCM-based therapies.

## 2 Methods

### 2.1 Botanical drugs and extracts

SP (batch No. SP-002) was collected from Guiyang (Guizhou, China), SG (batch No. SG-003) from Jinyun (Zhejiang, China), and SO (batch No. SO-005) from Ganzhou (Jiangxi, China). The herbal material samples were authenticated by the corresponding author, and the voucher specimens were deposited at the Institute of Chinese Medical Sciences, State Key Laboratory of Quality Research in Chinese Medicine, University of Macau. The extracts were prepared by extracting the samples with 50% ethanol at 60 °C for 1 h, followed by filtration and concentration under reduced pressure. The extracts were then lyophilized using a Virtis freeze dryer (The Virtis Company, New York, United States). The yields (%) of the resulting powdered were 21.6% (SG), 20.0% (SO), and 22.4% (SP), respectively.

### 2.2 Chemical characterization

The chemical profiles of SP, SO and SG extracts were characterized using a Waters ACQUITY-UPLC CLASS system (Waters Corp., Milford, United States) coupled with an ACQUITY UPLC BEH C18 column (150 mm × 2.1 mm, 1.8 μm) maintained at 40°C. The mobile phase was composed of A (0.2% phosphatic acid in water) and B (0.2% phosphatic acid in ACN), and the elution was performed with a gradient program of: 0–3 min, 8% B; 3–5 min, 8%–15% B; 5–8 min, 15%–18% B; 8–10 min, 18%–22% B; 10–15 min, 22%–33% B; 15–20 min, 33%–40%. The flow rate was 0.35 mL/min and the injection volume was 2 μL. The analytes were monitored at the UV wavelength of 215 nm. Prior to next injection, the column was washed with 100% B for 5 min and then equilibrated with the initial mobile phase for 10 min.

### 2.3 Cell culture and treatment

NCM460 and Caco-2 cells were obtained from the American Type Culture Collection (ATCC; Manassas, VA, United States), and maintained in McCoy’s 5A (Modified) and Dulbecco’s modified Eagle medium (DMEM), respectively, supplemented with 10% (v/v) fetal bovine serum (FBS) and 1% penicillin/streptomycin. Cells were incubated at 37 °C in a humidified atmosphere of 5% CO_2_ and 95% humidity. Cultures were maintained in 25 cm^2^ flasks (Thermo Fisher Scientific, MA, United States), with the medium refreshed every 2 days. Once cells reached approximately 85% confluence, they were washed, detached using a trypsin–EDTA solution (HyClone, United States), and collected in 15-mL centrifuge tubes (Thermo Fisher Scientific, MA, United States) for further analysis.

### 2.4 MTT assay

NCM460 and Caco-2 cells were inoculated into a 96-well plate (1 × 10^4^ cells/well) and allowed to adhere overnight. Cells were then treated with SH extracts at specified doses for 24 h, followed by incubation with medium containing 0.5 mg/mL MTT for an additional 3 h. The supernatant was discarded, and the formazan dye (dissolved in 150 μL DMSO) was added before absorbance was measured at 490 nm using a microplate reader (FlexStation3; Molecular Devices, United States).

### 2.5 ELASA assay

Blood samples were collected from the ocular region of mice and centrifuged at 1700 *g* and 4°C for 10 min to obtain serum. Colon tissues were lysed in a cell lysis solution (Beyotime, China), and proteins were extracted. Protein concentration was measured using a BCA protein assay kit (Thermo Fisher Scientific, MA, United States). Cytokine levels, including IL-6, IL-10, IL-17, and MCP-1, were quantified in serum and tissue lysates using a mouse ELISA kit (Beyotime, China) according to the manufacturer’s instructions.

### 2.6 Quantitative real-time PCR (qPCR) assay

Total RNA was extracted from cultured NCM460 cells and reverse transcribed into cDNA using a TaqMan Reverse Transcription Kit (Thermo Fisher, United States). cDNA amplification was performed using the Maxima SYBR Green qPCR Master Mix kit (Thermo Fisher Scientific, MA, United States). The primer sequences used in this study are listed below.

**Table:** 

Gene	Forward sequence	Reverse sequence
*IL-6*	AGA​CAG​CCA​CTC​ACC​TCT​TCA​G	TTC​TGC​CAG​TGC​CTC​TTT​GCT​G
*MCP-1*	AGA​ATC​ACC​AGC​AGC​AAG​TGT​CC	TCC​TGA​ACC​CAC​TTC​TGC​TTG​G
*IL-17*	CGG​ACT​GTG​ATG​GTC​AAC​CTG​A	GCA​CTT​TGC​CTC​CCA​GAT​CAC​A
*IL-10*	TCT​CCG​AGA​TGC​CTT​CAG​CAG​A	TCA​GAC​AAG​GCT​TGG​CAA​CCC​A
*H O -1*	CCA​GGC​AGA​GAA​TGC​TGA​GTT​C	AAG​ACT​GGG​CTC​TCC​TTG​TTG​C
*NQ O -1*	CCT​GCC​ATT​CTG​AAA​GGC​TGG​T	GTG​GTG​ATG​GAA​AGC​ACT​GCC​T
*GAPDH*	GTC​TCC​TCT​GAC​TTC​AAC​AGC​G	ACC​ACC​CTG​TTG​CTG​TAG​CCA​A

### 2.7 Western blotting

Total proteins were extracted from NCM460 cells using RIPA buffer supplemented with a protease inhibitor cocktail and 1 mM phenylmethanesulfonyl fluoride. Proteins (25–40 µg per sample) were separated by 10% or 12% sodium dodecyl sulfate polyacrylamide gel electrophoresis (SDS-PAGE) and transferred onto a polyvinylidene fluoride (PVDF) membrane (Bio-Rad, Hercules, CA, United States). The membrane was blocked with 5% skimmed milk in TBST (Tris-buffered saline, 0.1% Tween 20) for 1 h and subsequently incubated with primary antibodies against HO-1, NQO-1, Nrf2, Keap1, ZO-1, Occludin, and Claudin-1 (Cell Signaling Technology, United States). After washing, the membrane was incubated with secondary antibodies at room temperature for 2 h. Protein bands were detected using an enhanced chemiluminescence kit, captured with a Syngene Gel Imaging System (Bio-Rad), and quantified using Syngene software.

### 2.8 Trans-epithelial electrical resistance (TEER) measurement

Caco-2 cells were plated into a 12-well Transwell apical chamber. TEER (Ω cm^2^) was measured using an ERS-2 epithelial voltage meter (Merck Millipore, United States), as previously described (Xiong et al., 2021). When the epithelial resistance of filter-grown monolayers reached 500 Ω cm^2^, treatments were applied according to the experimental design, 3 weeks after confluence was reached. Each sample was measured three times (consecutively) to ensure consistency, and the values were corrected for background resistance due to the membrane insert.

### 2.9 Paracellular marker FD-4 (FITC-Dextran 4 kDa) flux measurements

Caco-2 monolayers were prepared as described above. Following treatment, cells were rinsed with phosphate-buffered saline (PBS) and incubated in the Transwell apical chamber for 2 h with Hank’s balanced salt solution containing 1 mg/mL FD-4 (Sigma-Aldrich, United States). A 100 µL sample was collected from the basolateral chamber, and FD-4 flux was measured. Fluorescence intensity was determined using a microplate reader (FlexStation3; Molecular Devices, United States) with excitation at 492 nm and emission at 520 nm.

### 2.10 Experimental animals and treatment

Male C57BL/6 mice (22–24 g) were obtained from the University of Macau’s Faculty of Healthy Science Animal Centre (Macau, China). The study was conducted under animal experimentation ethics approval number UMARE-016–2024. All mice were maintained on a standard laboratory diet with free access to water and housed under a 12-h light/dark cycle at 22°C ± 1°C with 50% relative humidity.

After a 1-week acclimation period, mice were randomly assigned to six groups: control (CTRL), DSS (3% DSS), Sulfasalazine (SASP, 400 mg/kg), SO (3 g/kg), SP (3 g/kg), and SG (3 g/kg), 6 mice per group. Body weight was measured daily and recorded. Once the most effective botanical drug species was identified, another batch of mice were subsequently divided into five groups for subsequent validation: CTRL, DSS (3% DSS), SPL (0.3 g/kg), SPM (1.5 g/kg), and SPH (3 g/kg), 6 mice per group.

### 2.11 Colon index and disease active index measurement

Mice were sacrificed by CO_2_ inhalation, and the colon was dissected. Colon length was measured, and the colon index (cm/g) was calculated as the ratio of colon length (cm) to body weight (g).

The DAI is calculated daily utilizing three parameters: weight loss, fecal consistency, and rectal bleeding. Every parameter receives a score ranging from 0 to 4. The overall DAI score is the aggregate of the scores for the three factors divided by three. Rectal bleeding was detected by occult blood in stool (OB) reagent (Baso, China).

### 2.12 Histopathology

Colon segments were fixed in 4% paraformaldehyde overnight, embedded in paraffin, and sectioned into 5-μm slices. The sections were stained with hematoxylin-eosin (H&E) for histological analysis.

### 2.13 Gut microbiota analysis

Fecal specimens were collected for gut microbiota analysis. Total DNA was extracted using a QIAamp-DNA Stool Mini Kit and assessed by electrophoresis on 1% agarose gels. The V3-V4 region of bacterial 16S rRNA genes was amplified using the Applied Biosystems ABI GeneAmp^®^ 9700 PCR System (Foster City, CA, United States). Amplification products were quantified with a QuantiFluor™-ST Handheld Fluorometer equipped with UV/Blue Channels (Promega Corporation, Madison, WI, United States).

Amplified 16S rRNA genes were sequenced using an Illumina MiSeq platform (Majorbio Bio-Pharm Technology Co., Ltd., Shanghai, China). After filtering and classification, operational taxonomic units (OTUs) with >97% similarity were identified. Representative OTU sequences were classified using the SILVA database and taxonomically categorized with the MOTHUR program. Gut microbiota analysis included Linear Discriminant Analysis Effect Size (LEfSe), principal coordinates analysis (PCoA), α diversity, β diversity, and Nonmetric Multi-Dimensional Scaling (NMDS) at the OTU level.

### 2.14 Statistical analyses

Data are presented as the means ± standard error of the mean (SEM). Comparisons between groups were performed using ANOVA and Student's *t*-tests in GraphPad Prism version 5.0 for Windows (GraphPad Software, United States). A significance threshold of *P* < 0.05 was applied. All experiments were conducted in triplicate to ensure repeatability.

## 3 Results

### 3.1 Chemical characterization of SH extracts

**FIGURE 1:**
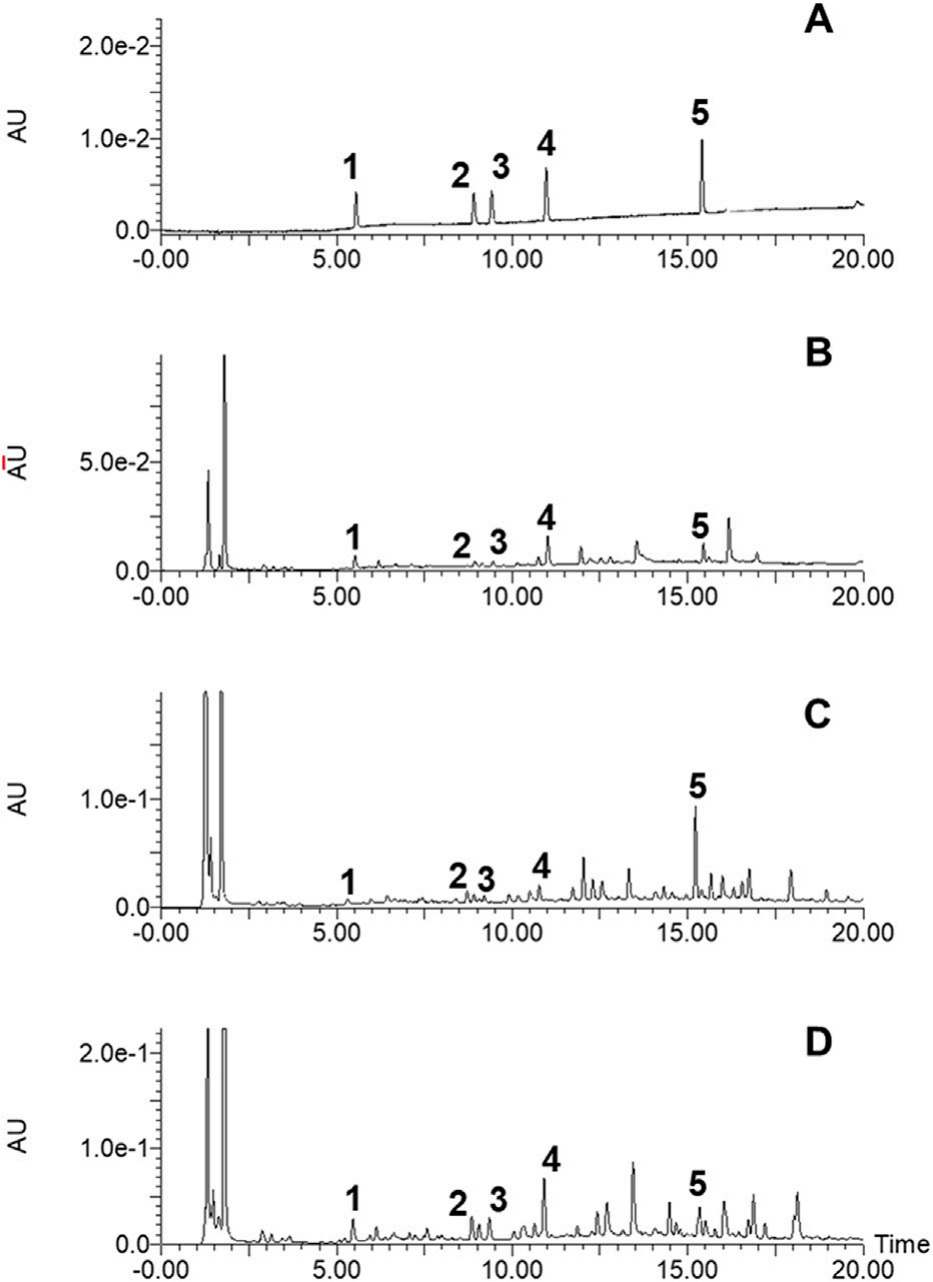
The UPLC-UV chromatograms of the mixed standards **(A)**, Sigesbeckia pubescens Makino (SP) extract **(B)**, Sigesbeckia orientalis L. (SO) extract **(C)**, and Sigesbeckia glabrescens Makino (SG) extract **(D)**. **1**: Chlorogenic acid; **2**: Rutin; **3**: Isoquercitrin; **4**: Isochlorogenic acid A; **5**: Kirenol.

The chemical profiles of SP, SO and SG extracts were performed using the UPLC-UV assay and the chromatograms of all samples were illustrated in Figure 1. SP presented a significant chemical profile while comparing to those of SO and SG, suggesting the potential difference in pharmacological activities for SP. Moreover, 5 chemicals in SP were further quantitively determined, and with the contents of 0.44% ± 0.01% (chlorogenic acid), 0.19% ± 0.01% (rutin), 0.05% ± 0.00% (isoquercitrin), 0.40% ± 0.02% (isochlorogenic acid A), and 1.16 ± 0.01 (kirenol), respectively.

### 3.2 SP is most effective against DSS-induced UC in mice

To evaluate the therapeutic effects of SH species in UC, we established a DSS-induced colitis model in mice and assessed key indicators of disease severity, including body weight loss, stool consistency, colon morphology, oxidative stress markers, and inflammatory cytokine levels. Comparisons were made between the three SH species (SP, SO, and SG) and the standard treatment, SASP.

**FIGURE 2:**
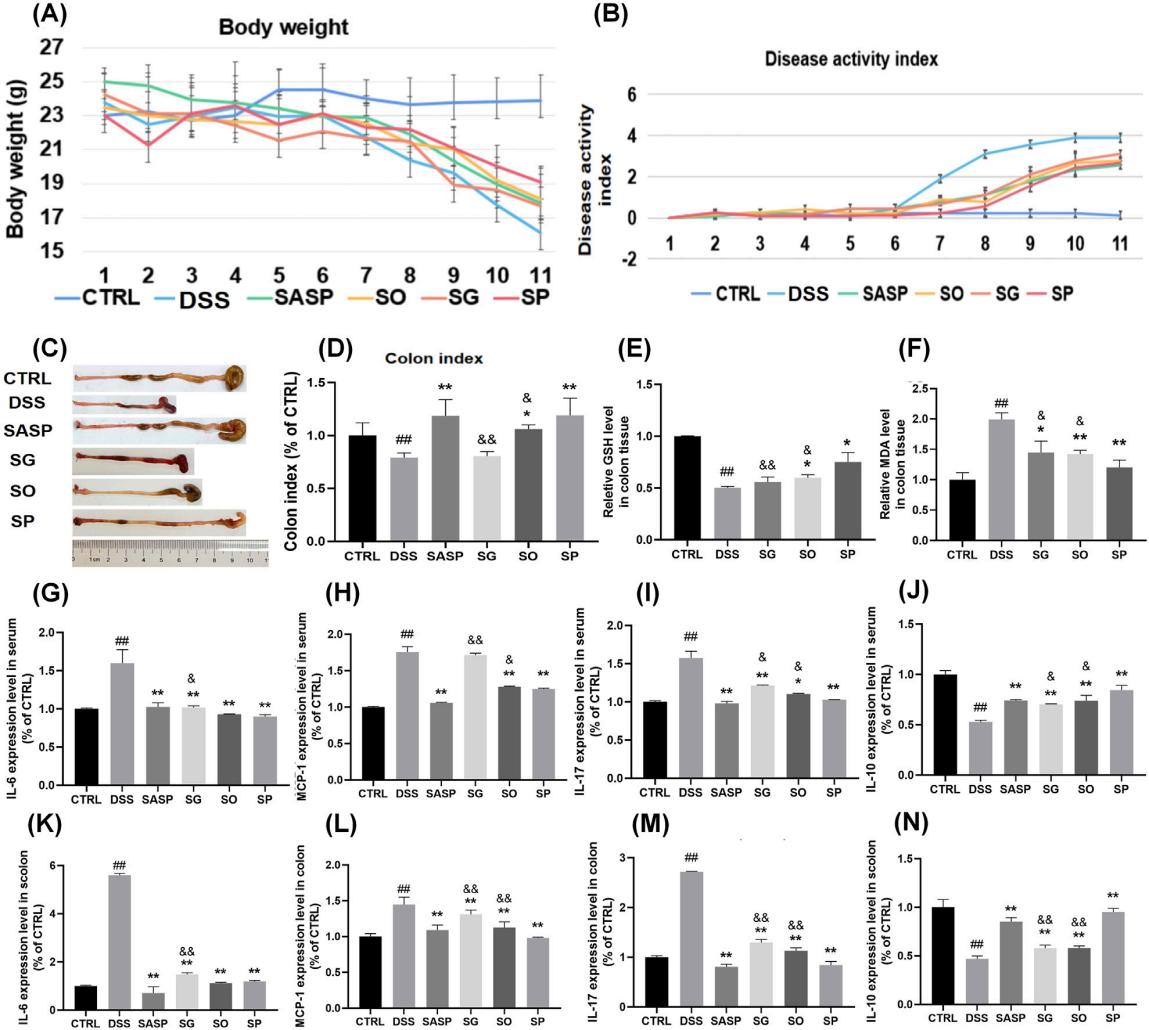
Effects of SH extracts on DSS-induced UC in mice. **(A)** Body weight changes over time. **(B)** Disease activity index (DAI). **(C)** Representative images of colons from each group. **(D)** Colon index (colon length/body weight). **(E–F)** Oxidative stress markers in colon tissue: **(E)** glutathione (GSH) levels and **(F)** malondialdehyde (MDA) levels. **(G–J)** Cytokine levels in serum: **(G)** IL-6, **(H)** MCP-1, **(I)** IL-17, and **(J)** IL-10. **(K–N)** Cytokine levels in colon tissue: **(K)** IL-6, **(L)** MCP-1, **(M)** IL-17, and **(N)** IL-10. Data are presented as mean ± SD (n = 6). CTRL, control; DSS, DSS model group (3% DSS); SASP, sulfasalazine (400 mg/kg); SO, *Siegesbeckia orientalis* L (3 g/kg); SP, *Siegesbeckia pubescens* Makino (3 g/kg); SG, *Siegesbeckia glabrescens* Makino (3 g/kg). ##*p* < 0.01 vs. CTRL; **p < 0.01 vs. MOD. andand *p* < 0.01 vs. SP. and *p* < 0.05 vs. SP.

We first noted that body weight remained stable in the CTRL group throughout the study, whereas a decline was observed in the DSS group starting on day 4 following DSS administration. By day 9, body weight in the DSS group had decreased significantly, reaching approximately 21% of the initial weight at day 11. Administration of SH extracts mitigated weight loss, with the least reduction observed in the SP group, followed by the SO, SASP, and SG groups (Figure 2A).

Stool frequency was notably higher in the DSS group compared to the CTRL group, with loose or watery feces containing mucus and/or blood. In addition, the DAI was significantly elevated in the DSS group relative to the CTRL group. Treatment with SASP, SP, SO, and SG significantly reduced DAI levels, with SP showing the most pronounced effect (Figure 2B).

Colon morphology was evaluated in each group after euthanasia of the mice (Figure 2C). A marked reduction in colon length was observed in the DSS group compared to the CTRL group. Colon index were SG 80.7%, SO 106.2%, SP 119.2% of CTRL group respectively. Treatment with SH extracts alleviated colon shortening to varying degrees, with SP demonstrating the most significant protective effect (Figure 2D). The measurements of glutathione (GSH) and malondialdehyde (MDA) is essential for evaluating oxidative stress and antioxidant capability, particularly relevant in inflammatory conditions like UC (Muro et al., 2024). GSH reflects endogenous antioxidant capacity (Aquilano et al., 2014). MDA reflects the degree of lipid peroxidation (Haro Girón et al., 2023). The level of GSH in colon tissue was lower in the DSS group than in the CTRL group, while MDA levels were elevated. Treatment with SH extracts also reversed these changes, suggesting that SH mitigates DSS-induced oxidative stress in the intestine. The most significant change was observed in the SP group relative to the DSS group, with a statistically difference compared to the SO and SG groups (Figures 2E, F).

Inflammatory cytokine levels were also affected by DSS-induced UC. IL-6, IL-17, and MCP-1 levels in both serum and colon tissue were significantly elevated following DSS administration and were subsequently reduced by treatment with SH extracts. This reduction was most pronounced in the SASP and SP groups. Conversely, IL-10 levels were significantly lower in both serum and colon tissue of DSS-induced UC mice compared to the CTRL group. Administration of SH extracts significantly increased IL-10 levels, with the SP group showing a greater effect than the SG and SO groups (*P* < 0.05) (Figures 2G–N).

Together, these findings indicate that SP is superior to SO and SG in treating DSS-induced UC. Given its strong therapeutic efficacy, SP was selected for further investigation to elucidate its underlying mechanism of action.

### 3.3 SP mitigates LPS-induced inflammation and oxidative stress in NCM460 cells

**FIGURE 3:**
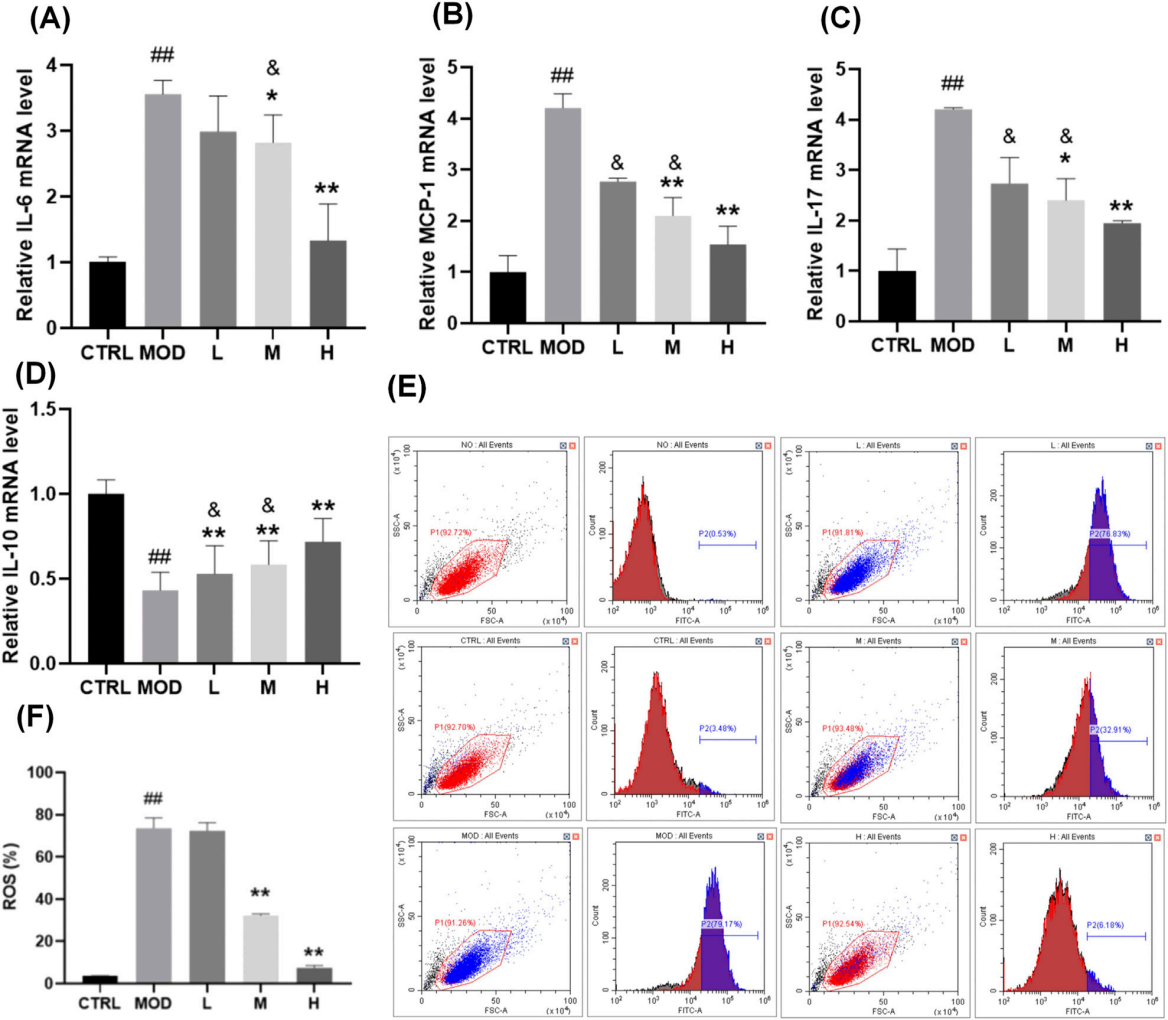
Effects of SP on LPS-induced inflammation and oxidative stress in NCM460 cells**. (A–D)** Relative mRNA expression levels of **(A)** *IL-6*, **(B)** *MCP-1*, **(C)** *IL-17*, and **(D)** *IL-10*, measured by qPCR. **(E–F)** Flow cytometry analysis **(E)** and quantification **(F)** of reactive oxygen species (ROS) levels in NCM460 cells following LPS stimulation and SP treatment. Data are presented as mean ± SD (n = 3). **CTRL**, control; **MOD**, LPS-treated group (100 ng/mL); **L**, low-dose *Siegesbeckia pubescens* Makino (40 μg/mL); **M**, medium-dose *Siegesbeckia pubescens* Makino (80 μg/mL); **H**, high-dose *Siegesbeckia pubescens* Makino (100 μg/mL). ##*p* < 0.01 vs. CTRL; **p < 0.01 vs. MOD. and *p* < 0.05 vs. SP.

To further validate the therapeutic mechanism of SP, *in vitro* investigations utilizing the human colonic epithelial cell line NCM460 were performed. This section primarily aimed to examine whether SP mitigates lipopolysaccharide (LPS)-induced inflammatory and oxidative damage, a critical pathological characteristic of UC, and to ascertain the role of the Nrf2/Keap1 signalling pathway in this mechanism. LPS, a strong inducer of pro-inflammatory and oxidative reactions, was utilized to replicate the UC microenvironment in colonic epithelial cells. It aimed to determine whether SP’s *in vivo* effectiveness is due to its direct control of epithelial cell homeostasis via the Nrf2/Keap1 axis by assessing its effects on inflammatory cytokines (IL-6, MCP-1, IL-17, and IL-10), reactive oxygen species (ROS) levels, and Nrf2 activation. The effects of SP on *IL-6*, *MCP-1*, *IL-17*, and *IL-10* secretion by qPCR were showed in Figures 3A–D. Consistent with the findings in the DSS-induced UC model, SH extracts mitigated inflammation induced by 100 ng/mL LPS, with SP demonstrating superior efficacy compared to SO and SG. Expression levels of *IL-6*, *MCP-1*, and *IL-17* increased following LPS stimulation but were significantly reduced by SH treatment, with the greatest reduction observed in the SP group. SP also further attenuated the LPS-induced reduction of *IL-10* levels.

To evaluate the protective effect of SP against LPS-induced oxidative stress, we measured ROS levels. NCM460 cells were exposed to 100 ng/mL LPS for 24 h, followed by treatment with SP at different doses. High and medium doses of SP significantly reduced ROS levels and conferred cytoprotection (Figures 3E, F).

### 3.4 SP regulates intestinal microecology and mucosal integrity

**FIGURE 4:**
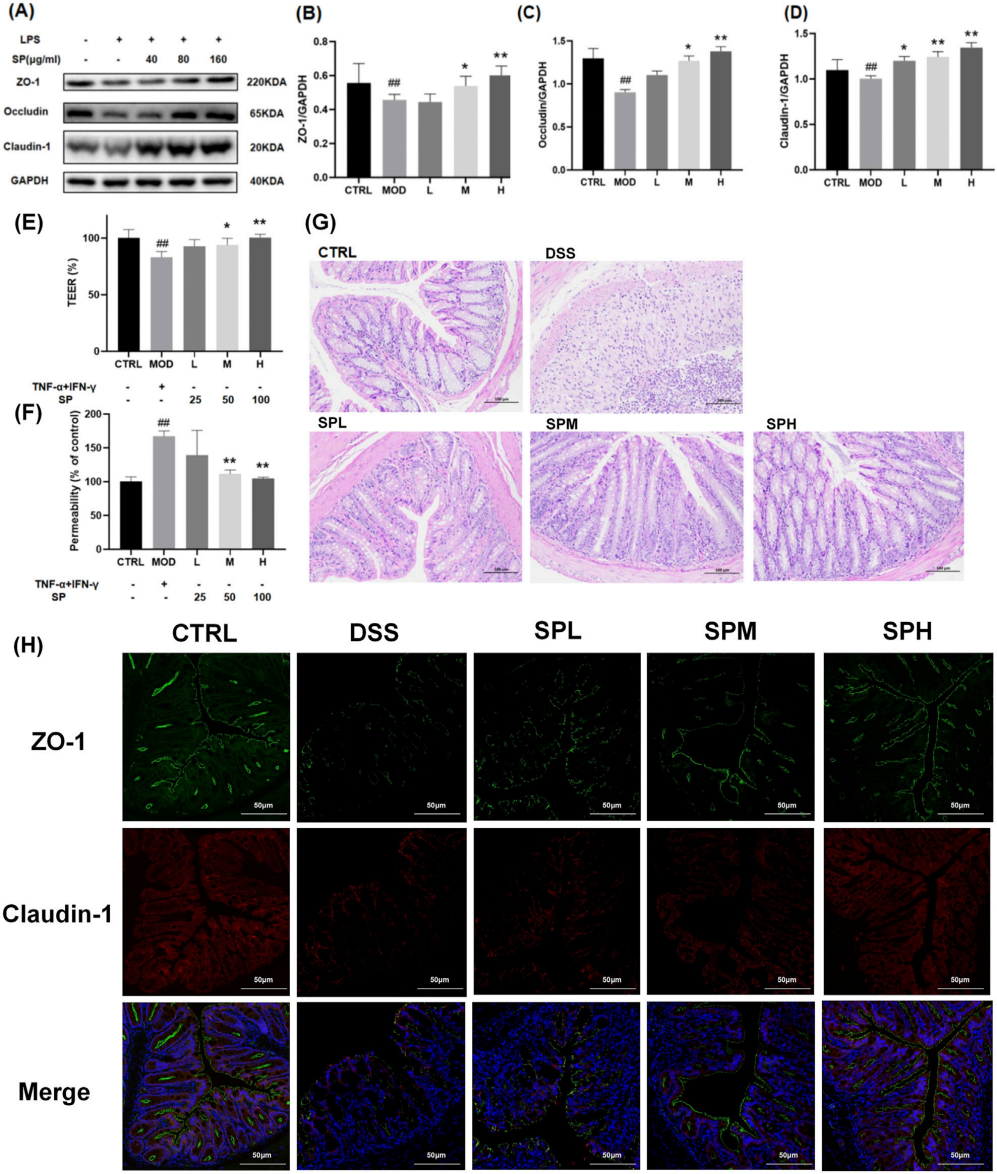
SP protects intestinal epithelial integrity by regulating tight junction proteins and reducing permeability**. (A)** Western blot analysis of ZO-1, Occludin, and Claudin-1 protein levels. **(B–D)** Relative protein expression levels of ZO-1, Occludin, and Claudin-1 measured by qPCR. **(E)** TEER analysis of Caco-2 monolayers after exposure to TNF-α and IFN-γ, with or without SP treatment. **(F)** Monolayer permeability assessed by FD-4 flux. **(G)** H&E staining of colon tissue sections isolated from different treatment groups. **(H)** Immunofluorescence analysis of ZO-1 and Claudin-1 expression in colon tissue. Scale bars: 100 μm. Data are presented as mean ± SD (n = 3). **CTRL**, control; **MOD**, LPS-treated group (100 ng/mL); **L**, low-dose *Siegesbeckia pubescens* Makino (40 μg/mL); **M**, medium-dose *Siegesbeckia pubescens* Makino (80 μg/mL); **H**, high-dose *Siegesbeckia pubescens* Makino (100 μg/mL); **DSS**, DSS model group (3% DSS); **SPL**, low-dose *Siegesbeckia pubescens* Makino (0.3 g/kg); **SPM**, medium-dose *Siegesbeckia pubescens* Makino (1.5 g/kg); **SPH**, high-dose *Siegesbeckia pubescens* Makino (3 g/kg). ##*p* < 0.01 vs. CTRL; **p < 0.01, *p < 0.05 vs. MOD.

UC is often associated with impaired epithelial barrier function, which contributes to diarrhoea through a leak-flux mechanism and exacerbates inflammation by increasing luminal antigen absorption (Tang et al., 2011). The intestinal epithelial barrier is maintained by intracellular junctional complexes, including tight junctions, adherens junctions, and desmosomes. Disruptions in tight junction proteins play a crucial role in UC pathogenesis (Groschwitz and Hogan, 2007). Zonula Occludens-1 (ZO-1), Claudin-1, and Occludin are essential components of tight junction proteins, directly implicated in the formation and stability of the junctional structure between intestinal epithelial cells. ZO-1 functions as a scaffolding protein that links transmembrane proteins (e.g., Claudin-1, Occludin) to the cytoskeleton, hence preserving the structural integrity of tight junctions. Claudin-1 modulates the selective permeability of paracellular channels and controls the passive diffusion of ions and small molecules. Occludin is involved in the regulation of the structural integrity of tight junctions and barrier function. After treating with LPS (100 ng/mL) ZO-1, Claudin-1, and Occludin expression was significantly downregulated. SP treatment mitigated this reduction, with the most pronounced effect observed in the SP-H group (Figures 4A–D).

Caco-2 monolayers derived from colon tissue, are widely used to assess intestinal integrity (Lopez-Escalera and Wellejus, 2022). We thus performed TEER and FD-4 flux assays to evaluate Caco-2 monolayer permeability. Following a 48-h exposure to 20 ng/mL TNF-α and 20 ng/mL IFN-γ, TEER levels dropped to approximately 20% of baseline (Figure 4E), while FD-4 flux increased to 1.5 times the baseline level (Figure 4F), indicating severe epithelial barrier dysfunction. Co-treatment with SP for 48 h improved TEER levels and reduced FD-4 flux in a dose-dependent manner, with the most significant protective effect observed at a dose of 100 μg (Figures 4E, F).

To further validate the protective role of SP, we analyzed colonic tissue from mice by H&E staining. The DSS group exhibited severe mucosal damage, including crypt shrinkage, localized necrosis, and inflammatory cell infiltration (Figure 4G). SP treatment reduced inflammatory infiltration and preserved crypt architecture, suggesting its protective effect against DSS-induced damage. The protective impact of SP on the structural integrity of the colon exhibited a dose-dependent relationship, yielding optimal outcomes at elevated dosages.

To examine the effects of SP on epithelial barrier function, we monitored ZO-1 and Claudin-1 protein expression by immunofluorescence staining. In the CTRL group, these proteins were expressed in a continuous, cohesive ring at the cell periphery, whereas only weak fluorescence signals were observed in the DSS group. SP treatment restored fluorescence intensity, indicating improved tight junction integrity (Figure 4H). Together, these findings demonstrate that SP protects against UC by preserving intestinal barrier function and mitigating mucosal injury.

### 3.5 SP regulates gut microbiota dysbiosis

**FIGURE 5:**
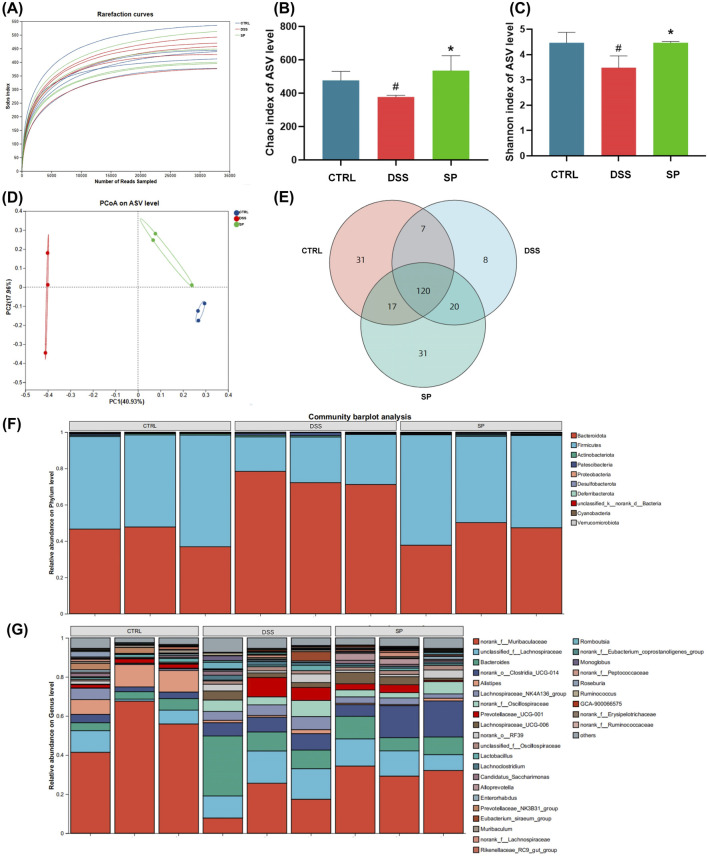
SP modulates gut microbiota composition in DSS-induced UC mice **(A)** Sobs rarefaction curves at the ASV level, showing sequencing saturation. **(B, C)** Alpha diversity analysis: **(B)** Chao index (community richness) and **(C)** Shannon index (community diversity). **(D)** Principal coordinate analysis (PCoA) of microbial beta-diversity based on Bray-Curtis dissimilarity, showing distinct clustering between groups. **(E)** Venn diagram illustrating differences in ASVs among the three groups **(F–G)** Microbial composition at different taxonomic levels: **(F)** phylum-level abundance and **(G)** genus-level abundance. The Sobs and Shannon indices were analyzed using the Wilcoxon rank-sum test. Data are presented as mean ± SD (n = 3). **CTRL**, control; **DSS**, DSS model group (3% DSS); **SPL**, low-dose *Siegesbeckia pubescens* Makino (0.3 g/kg); **SPM**, medium-dose *Siegesbeckia pubescens* Makino (1.5 g/kg); **SPH**, high-dose *Siegesbeckia pubescens* Makino (3 g/kg). #*p* < 0.05 vs. CTRL; *p < 0.05 vs. DSS.

As gut microbiota dysbiosis is closely associated with UC (Shen et al., 2018), we next evaluated microbial diversity in mice model. Mice feces were collected and sequenced to analyze the flora present. We generated an ASV (Sobs) rarefaction curve, showing that sequencing saturation was reached in all samples, confirming that the dataset was appropriate for further bioinformatics analysis. The number of sequenced ASVs in all three groups was sufficient to ensure the accuracy of the findings (Figure 5A).

From here, we could assess community richness and diversity using the Chao and Shannon indices, respectively. Both indices were lower in the DSS group compared to the CTRL group, indicating a loss of microbial diversity due to DSS treatment. SP administration reversed this effect, increasing both indices (Figures 5B, C). β-diversity, measured using Bray-Curtis dissimilarity and visualized with PCoA plots, revealed significant differences in microbial composition among the groups (Figure 5D). Venn diagram analysis further indicated that DSS-induced changes in microbial composition contributed to gut microbiota dysbiosis (Figure 5E). Notably, microbial profiles in the SP group clustered more closely with those in the CTRL group, suggesting a partial restoration of microbiota composition.

We analyzed the effects of SP on microbial composition at the phylum and genus levels (Figures 5F, G). At the phylum level, microbial profiles in the SP group closely resembled those in the CTRL group. An increase in *Bacteroidetes* is often associated with inflammatory bowel disease (IBD) progression, while the Firmicutes/Bacteroidetes (F/B) ratio plays a critical role in maintaining intestinal homeostasis (Stojanov et al., 2020). Reduced F/B ratios are frequently linked to IBD, and SP treatment appeared to restore this balance (Di Rosa et al., 2022). Interestingly, we saw that at the genus level, DSS treatment led to an increase in *Bacteroides* abundance and a decrease in *Muribaculaceae*. SP administration reversed these alterations, further suggesting a regulatory role in gut microbiota composition (Figure 5G). Together, these findings indicate that SP restores DSS-induced gut microbiota dysbiosis and modulates microbial composition at multiple taxonomic levels.

### 3.6 SP mitigates UC via activating the Nrf2/Keap1 pathway

UC is a chronic inflammatory bowel disorder marked by ongoing oxidative stress and dysregulated immune responses, resulting in mucosal injury and compromised intestinal barrier integrity. The Nrf2/Keap1 signaling pathway is crucial for sustaining redox homeostasis through the regulation of antioxidant enzymes (e.g., HO-1, NQO1) and detoxifying proteins. Under physiological settings, Nrf2 is confined in the cytoplasm by Keap1 and subjected to proteasomal degradation. Oxidative or electrophilic stress induces the separation of Nrf2 from Keap1, facilitating its nuclear translocation and the subsequent activation of cytoprotective genes. In UC, chronic inflammation and excessive generation of reactive oxygen species (ROS) surpass natural antioxidant defenses, leading to the inhibition of the Nrf2 pathway and exacerbated tissue damage. To investigate whether SP exerts its protective effects through this pathway, we analyzed the expression of Nrf2, Keap1, and their downstream targets in both LPS-stimulated NCM460 cells and DSS-induced UC mouse models.

**FIGURE 6:**
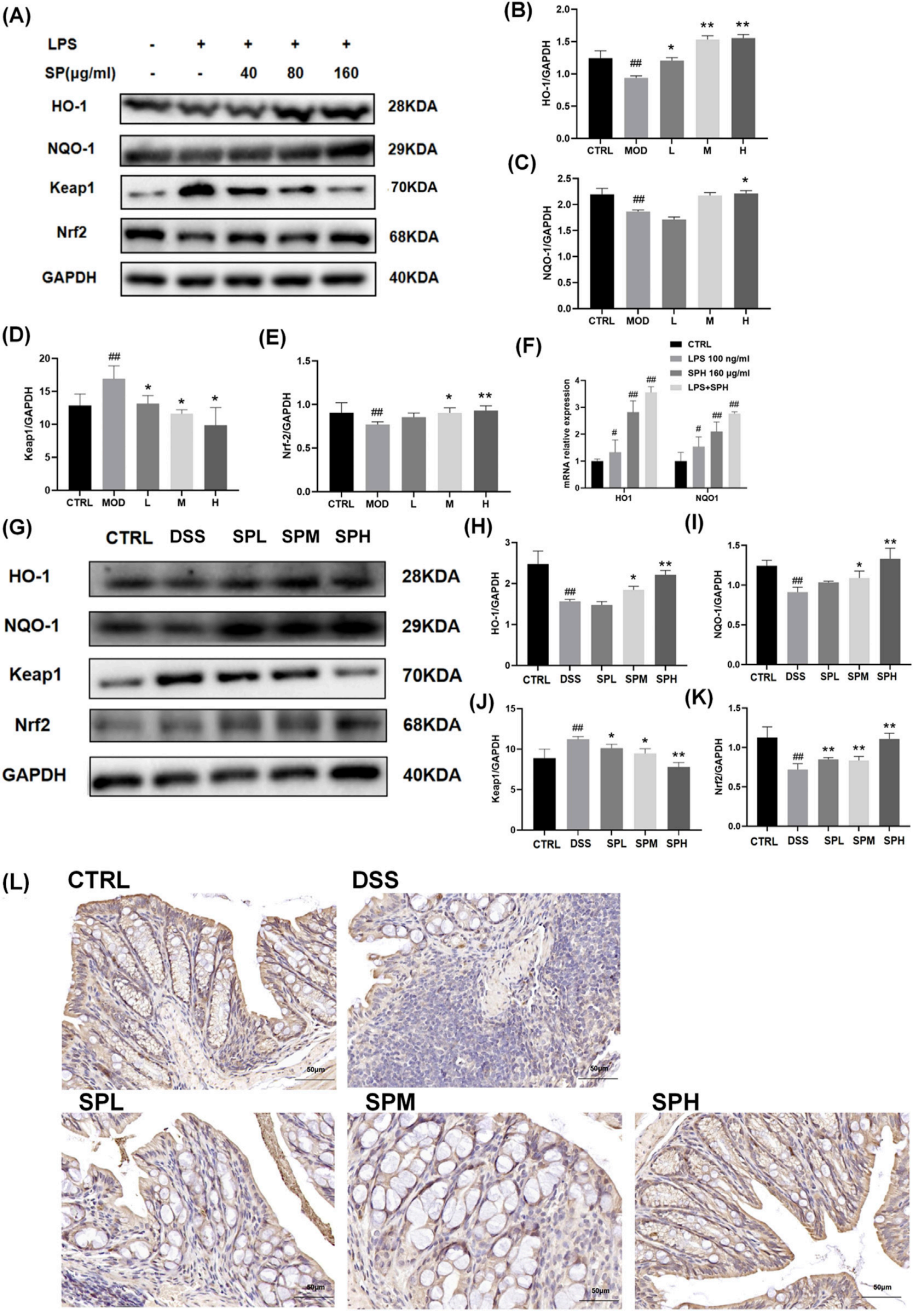
SP activates the Nrf2/Keap1 pathway in NCM460 cells and colon tissue **(A–E)** Western blot analysis of HO-1, NQO-1, Keap1, and Nrf2 expression in NCM460 cells. **(F)** mRNA expression levels of *H O -1* and *NQ O -1* in NCM460 cells. **(G–K)** Western blot analysis of HO-1, NQO-1, Keap1, and Nrf2 expression in colon tissue. **(L)** Immunohistochemical staining of Nrf2 in colon tissue. Scale bars: 50 μm. Data are presented as mean ± SD (n = 3). **CTRL**, control; **MOD**, LPS-treated group (100 ng/mL); **L**, low-dose *Siegesbeckia pubescens* Makino (40 μg/mL); **M**, medium-dose *Siegesbeckia pubescens* Makino (80 μg/mL); **H**, high-dose *Siegesbeckia pubescens* Makino (160 μg/mL). **DSS**, DSS model group (3% DSS); **SPL**, low-dose *Siegesbeckia pubescens* Makino (0.3 g/kg); **SPM**, medium-dose *Siegesbeckia pubescens* Makino (1.5 g/kg); **SPH**, high-dose *Siegesbeckia pubescens* Makino (3 g/kg). ##*p* < 0.01 vs. CTRL; **p < 0.01, *p < 0.05 vs. MOD or DSS.

Western blot and qPCR analyses revealed that Nrf2 expression was downregulated in NCM460 cells following LPS stimulation but significantly increased after SP treatment (Figures 6A–F). By contrast, Keap1 expression was lower 30.4% in the SPH-treated groups than in the MOD group. The expression of Nrf2 in the H group was 22.2% more than in the MOD group. A similar pattern was observed for HO-1 and NQO-1, which followed the expression trend of Nrf2. Consistent results were obtained in colon tissue from DSS-induced UC mice (Figures 6G–K): Nrf2 expression was markedly reduced 15% in the DSS group than CTRL group, whereas SP administration restored its levels, with the most pronounced effect observed in the SPH group, 20.6% higher in the SPH group compared to the DSS group. Additionally, SP upregulated the mRNA expression of Nrf2 downstream genes *H O -1* and *NQ O -1* in both LPS-stimulated and unstimulated NCM460 cells (Figure 6F). Immunohistochemical staining of Nrf2 in colon sections further supported these findings (Figure 6L). Together, these findings indicate that SP alleviates UC by activating the Nrf2/Keap1 pathway.

## 4 Discussion

UC is a significant clinical challenge, with current treatments often providing only partial symptom relief and leading to adverse effects. Identifying alternative therapeutic options that offer efficacy with fewer side effects is crucial. In contrast to synthetic pharmaceuticals that target a single route (e.g., 5-aminosalicylic acid or biologics), traditional Chinese medicines typically attain effectiveness through synergistic actions involving several plant metabolites and pathways. This multi-target property not only improves the capacity to cover the fundamental pathology of ulcerative colitis (oxidative-inflammatory-barrier imbalance), but also potentially mitigates drug resistance or adverse effects caused by excessive single-target inhibition, offering novel strategies for the long-term management of chronic inflammatory diseases. Historically SH has been recommended for treating arthritis, stroke, edema, and gastrointestinal disorders such as diarrhea and hematochezia. Modern clinical applications extend to acute enteritis. Given the potential of S*iegesbeckiae Herba* (SH), we investigated the therapeutic effects of SH extracts in DSS-induced UC and explored the underlying mechanisms.

We analyzed the effects of SH extracts on DSS-induced UC in mice by assessing multiple parameters, including body weight, DAI, colon length, and histological changes. As seen in clinical settings, weight loss and bloody stools were observed in DSS-treated mice, as well as a reduction in colon length. This reduction is associated with inflammation and edema of the colon, with edema increasing cellular fragility, rendering the epithelial cells susceptible to rupture and leading to intestinal ulcers. This process results in atrophy of the intestinal wall and shortening of the colon (Di Rosa et al., 2022). Moreover, histopathological analysis revealed that DSS-induced UC caused pronounced edema, hyperemia, epithelial shedding, crypt loss, and inflammatory cell infiltration in the colonic mucosa (Liu et al., 2023). Mice in the DSS group exhibited these pathological characteristics, whereas SP administration alleviated these symptoms and lowered the UC pathogenic score.

We further evaluated key inflammatory mediators involved in UC pathogenesis. IL-6 is a pro-inflammatory cytokine often linked to gastrointestinal malignancies (Zhang et al., 2024), with studies showing that its serum levels are significantly higher in IBD patients than in healthy individuals (Mudter and Neurath, 2007). We found that IL-6 expression was markedly increased in serum and colon tissue following DSS administration, whereas treatment with SH extracts reduced IL-6 levels. Among the three extracts, SP demonstrated the strongest suppression of IL-6, SP decreased by 78.8% compared to the DSS group in colon tissue, which was very close to the CTRL level. Similarly, MCP-1 expression was significantly elevated in UC lesions, with higher MCP-1 levels associated with greater disease severity (Elmahdy et al., 2022). DSS-treated mice showed increased MCP-1 expression, which was significantly reduced by SH treatment, particularly SP. MCP-1 has been implicated in UC pathogenesis, and studies have shown that animals lacking MCP-1 receptors exhibit fewer intestinal ulcers and reduced inflammation (Li et al., 2009). These findings indicate that SH extracts alleviate inflammation in UC, likely by suppressing IL-6 and MCP-1.

We also examined IL-17 and IL-10, both of which play critical roles in immune regulation. IL-17 is linked to host defense and chronic inflammatory responses, and elevated IL-17 levels are frequently observed in UC patients (Li et al., 2024). Consistently, we found that IL-17 expression was significantly increased in DSS-treated mice, while SH extracts, particularly SP, effectively suppressed IL-17 levels. Levels of IL-17 decreased by 69.1% in the SP group compared to the DSS group in colon tissue, equivalent to 84.4% of the CTRL group. By contrast, IL-10, an anti-inflammatory cytokine essential for maintaining intestinal homeostasis and mucosal integrity (Wang et al., 2021), was reduced in DSS-treated mice but restored following SH administration. The level of IL-10 in the SP group was 1.02 times higher than that in the DSS group and 0.95 times than that in the CTRL group, which was very close to the CTRL group. SP treatment resulted in the most pronounced IL-10 increase, reinforcing its role in protecting the intestinal mucosa.

Previous studies have demonstrated that gut microbial diversity is significantly reduced in UC patients (Matijašić et al., 2016). We observed a similar trend in DSS-treated mice, with lower Chao and Shannon indices indicating decreased microbial richness and diversity. SP treatment reversed this decline, suggesting a positive shift in gut microbiota composition. LEfSe analysis revealed that *Firmicutes* and *Bacteroidota* were dominant in the SP and DSS groups, respectively. SP also restored levels of *Muribaculaceae*, a major producer of short-chain fatty acids (SCFA) that play a key role in maintaining anti-inflammatory homeostasis (Zhu et al., 2024). A reduced Firmicutes/Bacteroidetes (F/B) ratio is a known feature of IBD (Stojanov et al., 2020), and SP treatment restored this balance. DSS exposure also led to increased *Bacteroides*, Prevotellaceae, and *unclassified_f_Lachnospiracea*, all of which have been associated with increased intestinal permeability and oxidative stress (Anderson et al., 2016; Li et al., 2021). SP administration also reversed these microbial alterations, supporting its role in regulating gut microbiota and alleviating UC symptoms. A bidirectional interaction between gut microbiota and the Nrf2/Keap1 pathway may underpin SP’s efficacy. On one side, SP-activated Nrf2 signaling augments antioxidant defenses, establishing a conducive milieu for beneficial microorganisms. In contrast, microbial metabolites such as SCFAs are recognized for their direct activation of Nrf2 (Liu et al., 2021), creating a synergistic loop. This interaction underscores SP’s multi-target efficacy in the therapy of UC. Future research may use metabolomics to delineate SP-induced alterations in microbial metabolites and utilize genetic models to corroborate the interaction between microbiota and Nrf2.

The intestinal mucosal barrier is essential for preventing the entry of harmful substances (Huang et al., 2022). Many studies have identified barrier dysfunction as an early feature of UC (Van Der Post et al., 2019). When barrier integrity is compromised, pathogenic bacteria can proliferate and release enterotoxins, increasing intestinal permeability and triggering immune dysregulation (Zou et al., 2021). This effect ultimately promotes chronic inflammation and worsens UC pathology. Improving intestinal mucosal barrier function is therefore considered a promising therapeutic strategy (Hu et al., 2021). As tight junction proteins have a critical role in barrier maintenance (Wang et al., 2016), we examined the expression of Claudin-1, Occludin, and ZO-1, which are essential for epithelial adhesion and integrity. Specifically, Claudin-1 and Occludin facilitate cell-to-cell adhesion, while ZO-1 links these proteins to the actin cytoskeleton, maintaining mucosal barrier function (Seo et al., 2021). We found that DSS exposure significantly downregulated these proteins, likely causing a weakening of the intestinal barrier. SP treatment reversed these changes, increasing the expression of tight junction proteins and reinforcing barrier integrity. Results from Transwell assays provided further confirmation, as TNF-α and IFN-γ exposure reduced epithelial resistance and increased permeability, both of which were restored following SP treatment. Similar improvements were observed in DSS-treated mice, where SP administration counteracted the DSS-induced decline in ZO-1 and Claudin-1 expression. These findings indicate that SP strengthens the intestinal barrier, a key factor in UC management.

There is also growing evidence that oxidative stress, resulting from an imbalance between oxidative and antioxidant systems, plays a key role in UC pathogenesis. An alkaloid called betaine can counteract oxidative stress by lowering oxidative stress markers, thereby slowing UC progression (Chen L. et al., 2022). The Chinese medicine SO can also enhance intracellular antioxidant defences, prevent cell death, support cell migration, and reduce inflammation, protecting non-cancerous cells from external oxidative damage (Chen C. et al., 2022). Here, we found that SP significantly inhibited the LPS-induced increase in ROS production, indicating a protective effect against oxidative stress.

To explore the underlying mechanism, we examined the Nrf2/Keap1 pathway, as it is a key regulator of cellular antioxidant defences (Linghu et al., 2024). SP administration significantly upregulated Nrf2 and its downstream genes *H O -1* and *NQ O -1* in both LPS-stimulated NCM460 cells and colon tissue from DSS-treated mice. As Nrf2 activation enhances cellular resilience against oxidative damage, these findings suggest that SP exerts its therapeutic effects by promoting antioxidant defenses through the Nrf2/Keap1 pathway.

This study systematically clarified the mechanism by which SP mitigates oxidative stress and inflammation via the Nrf2/Keap1 pathway using a combined investigation of *in vivo* (DSS-induced UC mice) and *in vitro* (NCM460 cells) scenarios. The disparities in efficacy across several SH species were unveiled for the first time, underscoring the significance of quality control in traditional Chinese medicine for clinical effectiveness. This integrates traditional Chinese medicinal knowledge with contemporary molecular mechanisms, establishing a framework for the modernization of traditional Chinese medicine. This study has disadvantages, including focusing on only SP crude extracts without isolating and identifying particular active plant metabolites. In addition to concentrating solely on the Nrf2 pathway, the interaction with other pathways (e.g., NF-κB, MAPK) was not investigated, thereby underestimating the multi-target characteristics of SP. Additionally, concerns exist regarding the limited duration of experiments and the differences in pathogenic processes and immunological microenvironments between animal models and human UC that require additional refinement.

## Data Availability

The datasets presented in this study can be found in online repositories. The names of the repository/repositories and accession number(s) can be found in the article/supplementary material.
